# Association between social participation and cognitive function among middle- and old-aged Chinese: A fixed-effects analysis

**DOI:** 10.7189/jogh.10.020801

**Published:** 2020-12

**Authors:** Yongjie Zhou, Zhuo Chen, Ian Shaw, Xiang Wu, Shiming Liao, Ling Qi, Lijuan Huo, Yifeng Liu, Ruoxi Wang

**Affiliations:** 1 Department of Rehabilitation, Shenzhen Kangning Hospital, Shenzhen, Guangdong, China; 2 College of Public Health, University of Georgia, Athens, Georgia, USA; 3 School of Economics, University of Nottingham Ningbo China, Ningbo, China; 4 School of Sociology and Social Policy, University of Nottingham, Nottingham, UK; 5 School of Medicine and Health Management, Tongji Medical College, Huazhong University of Science and Technology, Wuhan, Hubei, China; 6 School of Public Health, Fudan University, Shanghai, China; 7 School of Health Science and Nursing, Wuhan Polytechnic University, Wuhan, China; 8 Department of Psychiatry, The Affiliated Brain Hospital of Guangzhou Medical University (Guangzhou Huiai Hospital), Guangzhou, Guangdong, China; 9 Department of Psychiatry, Shenzhen Nanshan Center for Chronic Disease Control, Shenzhen, China; 10 Research Center for Rural Health Services, Hubei Province Key Research Institute of Humanities and Social Sciences, Wuhan, Hubei, China

## Abstract

**Background:**

Social Participation (SP) is known to benefit cognitive function. However, whether the positive relationship holds across different types of SP and dimensions of cognitive function, and whether the statement stays true in middle- and old-aged Chinese have not been investigated. The present study aimed to understand the current patterns of SP and cognitive function in China’s context, and therefore, explore the associations between cognitive function and SP at different levels from various aspects.

**Methods:**

A total of 7973 community residents aged 45 years and older were selected from the China Health and Retirement Longitudinal Study (CHARLS, 2011-2015). A fixed-effects analysis was used to explore the association between changes in SP (diversity, frequency, and type) and that in cognitive function (memory and mental status) over a four-year period.

**Results:**

Changing from no SP to more variety (β = 0.377, 95% confidence interval (CI) = 0.192-0.562 for 1 type, β = 0.703, 95% CI = 0.470-0.937 for ≥2 types) or higher frequency (β = 0.235, 95% CI = 0.007-0.462 for not regularly, β = 0.604, 95% CI = 0.411-0.798) of SP was associated with improvements in cognitive function. Playing mah-jong and using Internet were associated with improved memory but not with mental status. Sports and volunteering were associated with improved mental status but not with memory. The same pattern was observed in men and in women.

**Conclusions:**

The study confirmed that more diversity and higher frequency of SP was associated with improved cognitive function, whereas reminded policymakers to consider cultural context when developing target strategies to improve cognitive function.

Age-related cognitive impairment has become a global public health problem with the rapid population aging worldwide. Cognitive impairment causes function loss and dependency for those suffering from the disorders, poses a significant burden to their family [1], and brings tremendous challenges to the health care system and society [2]. Delving into novel approaches that could promote healthy brain aging with higher accessibility is crucial to address this problem, which is especially true to low- and middle-income countries, where a large number of residents are suffering from cognitive impairment but professional resources are limited [3].

In recent years, social participation (SP) has been regarded as a potentially effective approach considering its wide accessibility as well as its theoretical effect on cognitive function [4]. Prior studies have proposed three plausible mechanisms between SP and cognitive function. First, the ‘use it or lose it’ hypothesis suggests that participating in social activities may increase one’s cognitive activities that contribute to more mental stimulation and better brain function [5]. Second, the increased physical activities derived from taking part in social activities may help sustain cerebral blood flow, increase aerobic capacity and cerebral nutrient supply, thus lower the risk of cognitive impairment [6]. Third, SP may help one increase interpersonal interaction, obtain more social support, reduce the risk of suffering from psychological stress, and mitigate the stress-related neuronal changes that lead to cognitive decline [7].

Despite the broad agreement on the general benefit of SP on cognitive function [8], several knowledge gaps remain. First, since SP is an umbrella term that covers a broad range of activities, the relationship between a specific type of SP and cognitive function has not reached an agreement. For instance, a statistically significant association was observed between cognitive function and voluntary activities and interaction with friends in some studies [9,10] but not in others [11,12]. The underlying reason may be that the social significance of SP varies across cultural contexts [13], which calls for further investigation from an embeddedness perspective. Second, prior literature indicates that different types of SP translate to improved cognitive function through different paths [14]. For instance, internet use may contribute to better cognitive function through improved memory in information searching and processing [15]. Meanwhile, visiting friends involves increased social interactions [16]. The associated social support has been suggested to benefit both memory and executive functioning through reduced depressive symptoms [17]. However, the majority of the extant epidemiological studies treated cognitive function as a whole using scales such as Mini-Mental State Examination (MMSE) [11,18], leaving how the association holds across different dimensions of cognitive function in practice largely understudied. Such differences are critical for the development of targeted interventions. Third, most of the published studies only concerned observable variables, with unmeasurable individual-level confounding factors not controlled. Individual-level characteristics, such as gene, intelligence, and personality, may influence one’s health behaviours and mental health simultaneously [19,20]. Not considering the individual heterogeneity may lead to overestimation of the association or spurious correlation. Moreover, the vast majority of the limited evidence was from western countries, leaving what is happening in Chinese culture mostly unknown.

Under such circumstances, the present study has two aims: 1) to understand the patterns of SP and cognitive function in China’s context across a four-year period; 2) to explore the relationship between SP and cognitive function by digging down to specific types of SP and two dimensions of cognitive function. We hypothesised that: 1) in general, more types and higher frequency of SP is associated with improved cognitive function; and 2) the magnitude of association varies across different types of SP and different dimensions of cognitive function. We employed a fixed-effects analysis to address biases from omitted time-invariant variables. In light of the evident gender difference in China that strongly influences the SP behaviours and cognitive function in various ways [21], we further tested the association in subgroups.

## METHODS

### Sample and data collection

**Figure 1:**
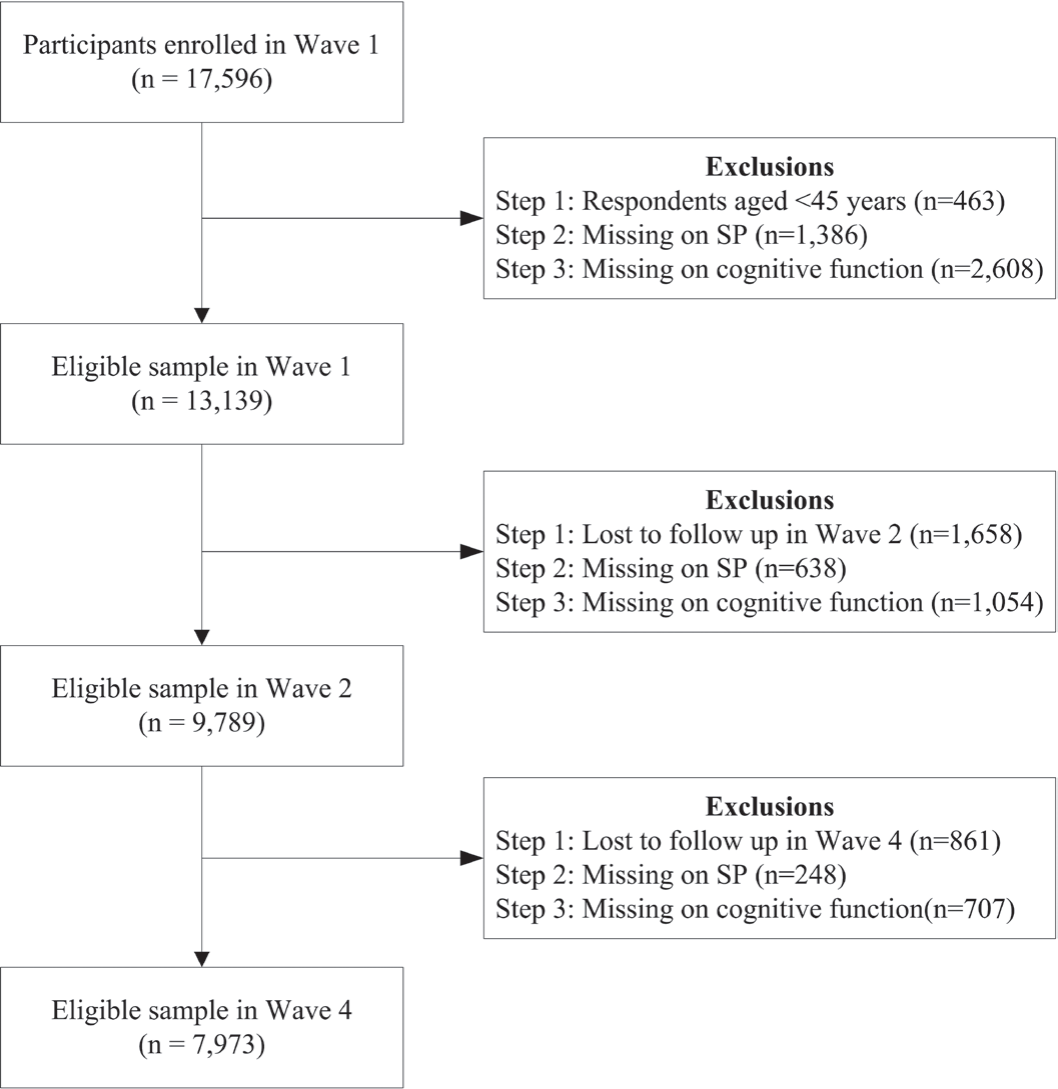
Flowchart of participant selection.

The data set used for statistical analysis in this study came from the China Health and Retirement Longitudinal Study (CHARLS). Underpinned by the multistage stratified probability proportional to size (PPS) sampling technique, the CHARLS research team surveys middle- and old-aged community-dwelling residents from 450 villages in 150 counties of 28 provinces in China. The baseline study was carried out in 2011 and involved 17 596 residents. Biannual follow-ups were conducted in 2013 and 2015. Considering that the fixed-effects regression examines the relationship between changes in independent and dependent variables across all three waves, a total of 7973 respondents were selected according to the selection process (**Figure 1**). We also implemented multiple imputation for missing data amongst those who participated in all three waves to check the robustness of our results, which yielded similar results (Table S1 in the **Online Supplementary Document**).

### Variables

#### Cognitive function

Similar to the US Health and Retirement Study, CHARLS employed components of the Telephone Interview of Cognitive Status (TICS) battery. Prior studies suggested measuring one’s cognitive function from two dimensions, including memory and a second factor labelled as ‘mental status’ [21,22]. Memory was measured by testing one’s skills relating to immediate word recall (0-10 points) and delayed word recall (0-10 points). Mental status was measured from three aspects, including orientation, visuoconstruction, and mathematical performance. Orientation (0-5 points) was measured by asking respondents to name today’s date, day of the week, and season; visuoconstruction was assessed by re-drawing a previously shown picture (0-1 point); mathematical performance (0-5 points) was measured by subtracting 7 from 100 consecutively for five times. We constructed the cognitive function from these two components (0-31 points) with higher scores suggesting better cognitive function.

#### Social Participation

We extracted seven types of SP from CHARLS, including 1) interaction with friends, 2) playing mah-jong or other board games, 3) going to sports or social clubs, 4) joining community-related organisations, 5) undertaking voluntary activities; 6) providing help to relatives or others without compensation, and 7) Internet use. For each type of SP, CHARLS [23] asked the respondent whether they had taken any of the aforementioned SP in the last month. If yes, they were further asked about the frequency accordingly (almost daily/ almost every week/ not regularly).

In this study, we examined SP from three aspects, namely type, maximum frequency, and diversity.

**Type:** since the proportion of those who took daily SP was less than 0.5%, we merged the two clusters ‘almost daily’ and ‘almost every week’ and recoded as ‘≥1/week’ for each type of SP. For the two variables ‘undertaking voluntary or charity activities’ and ‘providing help to relatives or others without compensation’, considering the small proportion of those who took these types of SP (less than 2%), we merged them to construct a new variable ‘voluntary activity’, following Lin’s [24] study. For ‘sports or social clubs’, ‘community organisations’, and ‘Internet use’, the proportion of the respondents with a frequency of once a week or higher was less than 1%. We then dichotomised them into No/Yes. **Maximum frequency**: the highest frequency of the aforementioned six types of SP, and was coded as: No/ not regularly/≥1/week. **Diversity**: we summed up the total number of types of SP and categorised it into: None/ 1 type/≥2 types.

#### Covariates

The confounding variables came from three aspects, including socio-demographic characteristics, health behaviours, health status-related variables, and time variable. For socio-demographic characteristics, we included gender, age, residency (rural, urban), education (illiterate, primary school and lower, junior middle school, senior middle school and higher), retirement (no, yes), marital status (single, partnered), living near children (no, yes), and percapital household income (Total household income (in US$)/ number of people in the household). For health behaviours, we considered current alcohol consumption (no, yes), and current smoking (no, yes). With respect to health status-related variables, we included numbers of types of non-communicable diseases (none, 1 type, 2 types, ≥3 types), and numbers of types of lower body constraints (none, 1 type, 2 types, ≥3 types). This study treated gender and residency as time-invariant variables, and the rest as time-varying variables.

### Data analysis

The overall aim of this study was to assess the association between SP and cognitive function with personal level time-invariant endogeneity addressed. We performed descriptive and multivariate analyses. First, frequencies and percentages were calculated for categorical data, whereas mean value and standard deviation (SD) were computed to describe normally-distributed continuous variables. χ^2^ test and *t* test were performed accordingly. Second, we employed correlation analysis and cross-sectional regression analysis to describe the relationship between SP and cognitive function. To test the correlation between SP and cognitive function, we conducted Spearman test for ordinal variables such as interacting with friends, mah-jong, and voluntary activities, whereas *t* test was employed for binomial variables such as sports, internet use, and community organisations. For the cross-sectional regression, we used pooled data from 2011, 2013, and 2015, and controlled for all time-varying and time-invariant variables. The results from both correlation and cross-sectional regression analysis indicated that the positive association holds across different types of SP and different dimensions of cognitive function (Table S2 in the **Online Supplementary Document**). Based on the results from the second stage analysis, we used a longitudinal linear fixed-effects regression model for further analysis. This model treats each individual as their own control and investigates how dependent variable changes as the independent variable changes. This, therefore, controls the potential individual-level time-invariant confounders, such as gene and personality, and helps us obtain reliable estimations regarding the relationship between SP and cognitive function.

Cognitive function*_it_* = μ_t_+β_1_SP_it_+β_2_x_it_+α_i_+ϵ_it_

Congitive function*_it_* indicates the cognitive performance for individual i at time t. SP_it_ suggests the diversity, frequency, and type of SP for individual i at time t. x_it_ denotes time-varying variables, such as health behaviours and health outcome variables. μ_t_ captures year-specific effects, whereas α_i_ characterises all time-invariant variables, such as gene and personality.

F-test and Hausman test were employed for model selection among ordinary least squares (OLS), random-effects model, and fixed-effects model. With a statistical significance (*P* < 0.001) for both tests, the results indicated that OLS or random-effects model would be biased, whereas the fixed effects model was preferred.

Prior studies have revealed that SP brings immediate influence to one’s mental health, which may fade away as time passes by [20]. In light of a two-year interval between two CHARLS follow-ups, we assessed the contemporaneous association between changes in SP and that in cognitive function. Coefficients (β) with 95% Confidence Intervals (95% CIs) were employed to measure the effect. Data were analysed using R Version 3.5.1 (R Foundation for Statistical Computing, Vienna, Austria).

### Ethics

The present study is a secondary analysis of the CHARLS public data, which was approved by the Ethical Review Committee of Peking University. This approval is updated annually.

## RESULTS

### Basic characteristics of the respondents

**Table 1** presents the basic characteristics of the respondents in 2011. Amongst the 7973 respondents, a greater proportion were men, rural residents, with primary school or lower education, living with a partner and near children, and currently working.

**Table 1:** Sample characteristics at baseline

Characteristics	All (n = 7973)		Men (n = 4156)		Women (n = 3817)	
	**n**	**%**	**n**	**%**	**n**	**%**
**Residency:**						
Urban	3065	38.44	1502	36.14	1563	40.95
Rural	4908	61.56	2654	63.86	2254	59.05
**Age:**						
Mean (SD)	57.79 (8.52)		58.7 (8.58)		56.8 (8.34)	
**Education:**						
Illiterate	1442	18.09	347	8.35	1095	28.69
≤Primary school	3474	43.58	1895	45.61	1579	41.37
Middle school	1969	24.70	1190	28.64	779	20.41
≥High school	1087	13.64	723	17.40	364	9.54
**Per capita household income:**						
Median (1^st^ quartile, 3^rd^ quartile)	805 (245, 1753)		754 (228, 1690)		857 (259, 1821)	
**Marital status:**						
Single	742	9.31	304	7.31	438	11.47
Partnered	7231	90.69	3852	92.69	3379	88.53
**Living near children:**						
No	642	8.24	376	9.33	266	7.07
Yes	7151	91.76	3656	90.67	3495	92.93
**Retirement:**						
No	6030	75.85	3336	80.56	2694	70.73
Yes	1920	24.15	805	19.44	1115	29.27
**Alcohol consumption:**						
No	5127	64.30	1756	42.25	3371	88.32
Yes	2846	35.70	2400	57.75	446	11.68
**Smoking:**						
No	5338	66.95	1742	41.92	3596	94.21
Yes	2635	33.05	2414	58.08	221	5.79
**Types of NCDs:**						
No NCD	2379	30.95	1336	33.27	1043	28.41
1 type	2203	28.66	1166	29.03	1037	28.25
2 types	1589	20.67	799	19.90	790	21.52
≥3 types	1516	19.72	715	17.80	801	21.82
**Lower body constraints:**						
No constraint	4112	51.57	2459	59.17	1653	43.31
1 type	1692	21.22	817	19.66	875	22.92
2 types	1117	14.01	466	11.21	651	17.06
≥3 types	1052	13.19	414	9.96	638	16.71

### Patterns of social participation and cognitive function

**Table 2** depicts SP and cognitive function of the respondents in all three waves. Nearly half of the respondents did not take any social activities in all three waves, especially in voluntary activities, sports or social clubs, Internet or community organisations. However, an upward trend of conducting social activities was observed in these four types of SP, and so as the diversity of SP. Cognitive function generally declined after four years. Nevertheless, there was an improvement in memory during 2011-2013. In general, women were less likely to participate in SP and were more disadvantaged in cognitive function than their counterparts in all three waves.

**Table 2:** Social participation and cognitive function in 2011-2015

	2011			2013			2015		
	**All**	**Men**	**Women**	**All**	**Men**	**Women**	**All**	**Men**	**Women**
**Social participation, %**									
**Diversity:**									
None	47.99	47.81	48.18	40.44	40.16	40.74	46.68	46.27	47.13
1 type	33.12	31.98	34.37	34.48	33.83	35.18	28.67	28.18	29.21
≥2 types	18.89	20.21	17.45	25.08	26.01	24.08	24.65	25.55	23.66
**Frequency:**									
None	47.99	47.81	48.18	40.44	40.16	40.74	46.68	46.27	47.13
Not regularly	14.76	15.66	13.78	16.71	18.14	15.14	16.33	17.81	14.72
≥1/week	37.25	36.53	38.04	42.86	41.70	44.12	36.99	35.92	38.15
**Interacting with friends:**									
None	63.68	65.76	61.41	58.20	60.76	55.41	63.90	64.89	62.82
Not regularly	10.67	10.76	10.58	13.71	13.76	13.65	13.24	13.81	12.63
≥1/week	25.65	23.48	28.01	28.09	25.48	30.94	22.85	21.29	24.55
**Mah-jong, cards, chess or other clubs:**									
None	78.65	74.76	82.89	76.50	71.78	81.63	78.74	74.88	82.94
Not regularly	9.01	10.71	7.15	8.98	11.21	6.55	8.42	10.25	6.42
≥1/week	12.34	14.53	9.96	14.52	17.01	11.82	12.84	14.87	10.64
**Voluntary activities:**									
None	91.36	90.64	92.14	84.87	84.53	85.25	83.46	82.24	84.78
Not regularly	6.18	6.81	5.50	10.81	11.45	10.11	12.32	13.19	11.37
≥1/week	2.46	2.55	2.36	4.31	4.02	4.64	4.23	4.57	3.85
**Sports or social clubs:**									
No	93.67	94.18	93.11	90.81	92.81	88.63	91.37	94.20	88.29
Yes	6.33	5.82	6.89	9.19	7.19	11.37	8.63	5.80	11.71
Internet:									
No	97.88	97.62	98.17	96.61	96.15	97.12	96.04	95.48	96.65
Yes	2.12	2.38	1.83	3.39	3.85	2.88	3.96	4.52	3.35
**Community organizations:**									
No	98.39	98.27	98.53	97.57	97.43	97.72	97.25	96.82	97.72
Yes	1.61	1.73	1.47	2.43	2.57	2.28	2.75	3.18	2.28
**Cognition, mean (SD)**									
Total score	15.41 (4.87)	15.99 (4.54)	14.78 (5.13)	15.32 (5.11)	15.91 (4.68)	14.68 (5.49)	14.59 (5.26)	15.02 (4.91)	14.12 (5.59)
Memory	7.49 (3.26)	7.48 (3.17)	7.49 (3.35)	7.55 (3.40)	7.56 (3.27)	7.53 (3.54)	7.00 (3.52)	6.89 (3.41)	7.14 (3.62)
Mental status	7.93 (2.71)	8.51 (2.43)	7.29 (2.86)	7.78 (2.78)	8.34 (2.50)	7.16 (2.94)	7.58 (2.80)	8.13 (2.53)	6.99 (2.97)

SD – standard deviation

### Association between SP and cognitive function

The associations between changes in SP and that in the total cognitive function, memory, and mental status across three waves are presented in **Table 3**. Transmitting from no SP to more varieties or a higher frequency of SP was strongly associated with an improved cognitive function. The general association held across dimensions of cognitive function and gender subgroups. With the exception of joining community organisations, taking part in the other five types of SP was positively associated with a better cognitive function, amongst which, the association was most prominent for playing mah-jong.

**Table 3:** Associations between social participation and cognitive function

	Total score			Memory			Mental status		
	**All**	**Men**	**Women**	**All**	**Men**	**Women**	**All**	**Men**	**Women**
	**β (95% CI)**	**β (95% CI)**	**β (95% CI)**	**β (95% CI)**	**β (95% CI)**	**β (95% CI)**	**β (95% CI)**	**β (95% CI)**	**β (95% CI)**
**Variety (Ref: None)**									
1 type	0.377*** (0.192, 0.562)	0.355* (0.080, 0.629)	0.404** (0.154, 0.654)	0.248*** (0.104, 0.391)	0.208+ (-0.003, 0.419)	0.291** (0.096, 0.487)	0.129* (0.026, 0.233)	0.147+ (-0.007, 0.300)	0.113 (-0.028, 0.254)
≥2 types	0.703*** (0.470, 0.937)	0.522** (0.178, 0.865)	0.856*** (0.537, 1.175)	0.440*** (0.258, 0.622)	0.321* (0.056, 0.586)	0.541*** (0.292, 0.791)	0.263*** (0.130, 0.395)	0.201* (0.008, 0.394)	0.315*** (0.135, 0.494)
**Frequency (Ref: None)**									
Not regularly	0.235* (0.007, 0.462)	0.075 (-0.254, 0.405)	0.378* (0.062, 0.693)	0.119 (-0.058, 0.296)	-0.030 (-0.284, 0.223)	0.260* (0.014, 0.507)	0.115+ (-0.013, 0.243)	0.106 (-0.079, 0.290)	0.117 (-0.060, 0.295)
≥1/week	0.604*** (0.411, 0.798)	0.615*** (0.325, 0.904)	0.610*** (0.351, 0.869)	0.407*** (0.257, 0.557)	0.416*** (0.193, 0.639)	0.413*** (0.211, 0.616)	0.197*** (0.089, 0.306)	0.199* (0.036, 0.361)	0.197** (0.051, 0.343)
**Interacting with friends (Ref: None)**									
Not regularly	0.097 (-0.144, 0.338)	0.001 (-0.356, 0.356)	0.175 (-0.151, 0.502)	0.055 (-0.132, 0.242)	-0.018 (-0.292, 0.257)	0.113 (-0.142, 0.368)	0.042 (-0.093, 0.177)	0.018 (-0.182, 0.218)	0.063 (-0.120, 0.246)
≥1/week	0.518*** (0.324, 0.712)	0.574*** (0.278, 0.870)	0.490*** (0.232, 0.749)	0.317*** (0.166, 0.468)	0.386*** (0.158, 0.614)	0.277** (0.075, 0.479)	0.201*** (0.091, 0.310)	0.188* (0.022, 0.354)	0.214** (0.068, 0.359)
**Mah-jong, cards, chess or other clubs (Ref: None)**									
Not regularly	0.240 (-0.078, 0.558)	0.204 (-0.227, 0.635)	0.299 (-0.172, 0.769)	0.145 (-0.102, 0.392)	0.113 (-0.219, 0.444)	0.199 (-0.168, 0.567)	0.095 (-0.084, 0.273)	0.091 (-0.151, 0.333)	0.099 (-0.165, 0.364)
≥1/week	0.640*** (0.326, 0.954)	0.52* (0.086, 0.963)	0.770*** (0.321, 1.219)	0.589*** (0.345, 0.833)	0.432* (0.094, 0.769)	0.755*** (0.404, 1.106)	0.051 (-0.126, 0.227)	0.093 (-0.153, 0.339)	0.015 (-0.238, 0.267)
**Voluntary activities (Ref: None)**									
Not regularly	0.376** (0.121, 0.632)	0.175 (-0.188, 0.538)	0.557** (0.197, 0.917)	0.230* (0.031, 0.428)	0.096 (-0.184, 0.375)	0.349* (0.068, 0.630)	0.147* (0.003, 0.290)	0.079 (-0.124, 0.283)	0.209* (0.006, 0.411)
≥1/week	0.358+ (-0.054, 0.770)	0.675* (0.071, 1.279)	0.109 (-0.455, 0.673)	0.154 (-0.166, 0.474)	0.376 (-0.089, 0.841)	-0.018 (-0.459, 0.422)	0.204+ (-0.027, 0.436)	0.299+ (-0.040, 0.637)	0.127 (-0.190, 0.444)
**Sports or social clubs (Ref: No)**									
Yes	0.362* (0.042, 0.681)	0.030 (-0.476, 0.537)	0.530* (0.123, 0.938)	0.161 (-0.087, 0.409)	0.020 (-0.368, 0.409)	0.229 (-0.090, 0.548)	0.201* (0.022, 0.380)	0.010 (-0.274, 0.294)	0.301** (0.072, 0.531)
**Internet (Ref: No)**									
Yes	0.644* (0.013, 1.273)	0.731+ (-0.068, 1.530)	0.549 (-0.470, 1.568)	0.506* (0.017, 0.995)	0.534+ (-0.081, 1.149)	0.512 (-0.284, 1.308)	0.138 (-0.216, 0.491)	0.197 (-0.250, 0.645)	0.037 (-0.536, 0.609)
**Community organisations (Ref: No)**									
Yes	0.421 (-0.098, 0.939)	0.159 (-0.572, 0.889)	0.702+ (-0.033, 1.437)	0.088 (-0.314, 0.491)	0.036 (-0.526, 0.599)	0.159 (-0.414, 0.734)	0.333* (0.042, 0.624)	0.122 (-0.287, 0.532)	0.542** (0.130, 0.955)

CI – confidence interval

*All models controlled time-varying variables, including year, education, marital status, living near children, retirement status, percapita household income, alcohol consumption, smoking, # of types of NCDs, and # of types of lower body constraints; + *P* < 0.1, * *P* < 0.05, ** *P* < 0.01, *** *P* < 0.001.

The association between SP and cognitive function varied across types of SP and between different dimensions of cognitive function (**Table 3**). For interacting with friends and voluntary activities, taking part in these two types of SP was associated with an improved memory as well as mental status. Meanwhile, doing sports was only associated with improved mental status but not memory. On the contrary, for mah-jong and Internet use, a positive association was only observed in their relationship with memory but not mental status. No significant interaction effect with gender was found in any aspect of SP.

## DISCUSSION

Using three waves of a longitudinal survey, this study examined the association between changes in SP and that in cognitive function among middle- and old-aged Chinese with the unobservable personal characteristics related variables controlled. This study has yielded four main findings: 1) a general downward trend was observed in all three dimensions of cognitive function with, however, improvement in memory function during 2011-2013; 2) the prevalence of SP varied across different types of SP, and an upward trend was observed in some activities; 3) more variety, higher frequency of SP was correlated with both better memory and mental status, whereas, the relationship varied across specific types of SP and between different dimensions of cognitive function; and 4) Gender-disparity was observed in the patterns of SP and cognitive function, but the association between SP and cognitive function remained similar in gender subgroups.

### Changes of cognitive functions

In this study, we found that the cognitive function of middle- and old-aged respondents generally declined as they age. Supported by prior studies [25,26], this finding indicates that the threat of cognitive impairment may become more severe in the context of population ageing in China. Meanwhile, as we observed improvement in memory during 2011-2013, we noted that the trajectory was nonlinear, which is in line with some studies [10,11]. This finding reveals that it is still viable to improve one’s cognitive function with properly developed interventions even in their middle- and old-age. This reminds the value of identifying preventive approaches that would enable more people to enjoy their late-life with high quality [27].

### Patterns of social participation

As a means with the potential to contribute to one’s cognitive function, the prevalence of SP was significantly lower in older Chinese compared with their counterparts in developed countries [20]. More interestingly, compared with western settings where community associations [28] and sport or social clubs [11,20] are more popular, mah-jong and interaction with friends were observed with higher prevalence amongst older Chinese. This echoes our proposition that cultural context should be taken into consideration when investigating the patterns of SP and its association with cognitive function. It is inspiring to observe a general uptrend of SP prevalence, such as diversity of SP and voluntary activities. This indicates that one’s social participation behaviour is intervene-able as long as the SP-friendly environment is well established.

### Association between social participation and cognitive function

Consistent with our first hypothesis, this study found a significantly positive association between changes in diversity and frequency in SP and that in cognitive function. The finding is also in line with prior studies [19,29]. Moreover, we found that this relationship held across different dimensions of cognitive function and gender subgroups, which highlights the role of SP in predicting or even promoting cognitive function among middle- and old-aged Chinese [21].

Our results supported our second hypothesis that the strength of the association mainly depended on the type of activity and the dimension of cognitive function. We found that interacting with friends and voluntary activities were associated with better cognitive function in both memory and mental status; playing mah-jong and Internet use were only associated with better memory; whereas joining sports or social clubs was only associated with better mental status. A handful of prior studies have documented the type-related variations in this relationship [9,30], but few further investigated the relationship across different dimensions of cognitive function nor clarified the underlying mechanism.

With the underlying mechanism not fully comprehended, we speculate that different types of SP stimulate different parts of the brain with unique functions [31]. For instance, playing mah-jong requires attention and memory [32], and one’s fine motor movement and eye-hand coordination may be improved when playing mah-jong [33], which could contribute to enhancing brain function regarding digit forward and verbal memory [34]. This is similar for Internet use, which consists of novel and complex mental processes for older adults, especially requiring memory [15]. However, both mah-jong and Internet use tend to be indoor activities, whilst the part of the brain activated by them may not be closely related to time orientation or visuoconstruction. In contrast, sports tend to be outdoor activities in China. In other words, these types of activities may improve physiological functionality and stimulate the part of the brain that is associated with tasks involving time orientation, visuospatial [35], and mathematic performance [36]. Interacting with friends [16] and voluntary activities [37] were found to be associated with more extensive social networks and less depressive symptoms, which may contribute to better cognitive function via mitigating stress-related impact. Besides, the information exchange derived from inter-personal communication may help form healthy behaviours and have a more far-reaching influence on better cognitive performance [38]. As two types conventional SP in China, the identification of a significant association between cognitive function and mah-jong and interacting with friends suggested that policies to promote moderate mah-jong or interaction with friends may be worth consideration in light of its wide popularity [16]. Similarly, the growing prevalence of sports, such as square dance and Tai-chi [39], calls for special attention in policymaking.

### Gender differences

Another interesting finding is the significant gender difference in the patterns of cognitive function and SP, but not in the relationship between these two. Women residents suffered from poor cognitive function and fewer chances for social activities when compared with their counterparts, which is consistent with prior studies [18,21]. This indicates that older women are in higher need for targeted SP to prevent cognitive impairment.

Prior findings regarding the gender-disparity in the relationship between SP and mental health are competing, as some reporting favourable effects only in women [40] or only in men [41], whereas some observed no gender differences [42]. The present study observed similar patterns in gender subgroups, and thus suggested that encouraging sufficient SP may be equally beneficial to men and women in China’s context. On the one hand, the finding echoes Tomioka et al. [19] who reminded the need to understand the relationship between SP and cognitive function in specific contexts. On the other hand, by acknowledging the complex mechanisms that underlie SP’s influence on cognitive function, this study calls for future studies to test whether there is a gender-difference in specific paths, such as through neurological health or mental health [43], with more sophisticated research design.

### Strength and limitations

To the best of our knowledge, the present study is the first to examine the association between SP and cognitive function in China while ruling out the potential endogeneity that derives from personal characteristics such as gene and personality. Meanwhile, this study is one of the very few studies that further investigate the relationship in different dimensions of cognitive function and across different gender groups. These findings confirmed that more diversity and higher frequency of SP associated with better cognitive function in both men and women, suggested that conventional SP such as mah-jong or interacting with friends may be worth special attention, and also informed the type of SP that predicts improvements in the specific function of the brain in China’s context, which has implications for the development of targeted interventions.

The present study has several limitations. First, the study participants may not have fully represented the middle- and old-aged population in China, as the CHALS team conducted the survey among community-dwelling residents, whereas, those who live in institutions such as nursing home were not included. Second, information regarding participation in social activities was self-reported. The recall bias may result in underestimation of the SP behaviours due to hypomnesia in one’s older age. Third, this study assessed the contemptuous association between SP and cognitive function rather than the lagged effect, considering that SP brings contemporaneous effects to mental health that may diminish over time [20]. The causal relationship awaits further investigation. Fourth, the fixed-effects model has controlled individual-level time-invariant characteristics, nevertheless, there might be other unmeasurable or unmeasured time-varying variables not been included in this study. Fifth, the sample size for those who used Internet or joined community organisations were too small to be further categorised according to frequency. We delegate stratified analysis to future studies with a larger sample size.

## CONCLUSIONS

Underpinned by a nationwide representative survey, we observed the patterns of SP behaviours and cognitive function in middle- and old-aged Chinese, and further explored the association between changes in SP and that in cognitive function by examining three dimensions of SP and two dimensions of cognitive function. The present study mainly revealed that: 1) more variety and higher frequency of of SP was generally associated with improved cognitive function, whereas the magnitude of the relationship depended on the specific type of SP and the dimension of cognitive functions; and 2) gender-disparity was observed in the patterns of SP and cognitive function, but the association between SP and cognitive function remained similar in gender subgroups.

## Additional material

Online Supplementary Document

## References

[1] Frankish H Horton R Prevention and management of dementia: a priority for public health. Lancet. 2017;390:2614-5. 10.1016/S0140-6736(17)31756-7 28735854

[2] World Health Organization. Global action plan on the public health response to dementia (2017-2025). Geneva: World Health Organization; 2017.

[3] World Health Organization. Mental Health Workers in China 2017. Geneva: World Health Organization; 2017.

[4] Xu H, Vorderstrasse AA, McConnell ES, Dupre ME, Ostbye T, Bei W. Migration and cognitive function: a conceptual framework for Global Health Research. Global Health Research and Policy. 2018;3. 10.1186/s41256-018-0088-5 PMC6267896 30519639

[5] Livingston G Sommerlad A Orgeta V Costafreda SG Huntley J Ames D Dementia prevention, intervention, and care. Lancet. 2017;390:2673-734. 10.1016/S0140-6736(17)31363-6 28735855

[6] Laurin D Verreault R Lindsay J MacPherson K Rockwood K Physical activity and risk of cognitive impairment and dementia in elderly persons. Arch Neurol. 2001;58:498-504. 10.1001/archneur.58.3.498 11255456

[7] Kremen WS Lachman ME Pruessner JC Sliwinski M Wilson RS Mechanisms of Age-Related Cognitive Change and Targets for Intervention: Social Interactions and Stress. J Gerontol A Biol Sci Med Sci. 2012;67:760-5. 10.1093/gerona/gls125 22570134 PMC3391069

[8] Bourassa KJ Memel M Woolverton C Sbarra DA Social participation predicts cognitive functioning in aging adults over time: comparisons with physical health, depression, and physical activity. Aging Ment Health. 2017;21:133-46. 10.1080/13607863.2015.1081152 26327492

[9] Fu C Li Z Mao Z Association between Social Activities and Cognitive Function among the Elderly in China: A Cross-Sectional Study. Int J Environ Res Public Health. 2018;15:231. 10.3390/ijerph15020231 29385773 PMC5858300

[10] Chiao C Beyond health care: Volunteer work, social participation, and late-life general cognitive status in Taiwan. Soc Sci Med. 2019;229:154-60. 10.1016/j.socscimed.2018.06.001 29908766

[11] Hwang J Park S Kim S Effects of Participation in Social Activities on Cognitive Function Among Middle-Aged and Older Adults in Korea. Int J Environ Res Public Health. 2018;15:2315. 10.3390/ijerph15102315 30347887 PMC6210154

[12] Su X Huang X Jin Y Wan S Han Z The relationship of individual social activity and cognitive function of community Chinese elderly: a cross-sectional study. Neuropsychiatr Dis Treat. 2018;14:2149-57. 10.2147/NDT.S160036 30197518 PMC6113942

[13] Chiao C Weng L Botticello AL Social participation reduces depressive symptoms among older adults: An 18-year longitudinal analysis in Taiwan. BMC Public Health. 2011;11:292. 10.1186/1471-2458-11-292 21569285 PMC3103460

[14] Kelly ME Duff H Kelly S Power JEM Brennan S Lawlor BA The impact of social activities, social networks, social support and social relationships on the cognitive functioning of healthy older adults: a systematic review. Syst Rev. 2017;6:259. 10.1186/s13643-017-0632-2 29258596 PMC5735742

[15] Small GW Moody TD Siddarth P Bookheimer SY Your Brain on Google: Patterns of Cerebral Activation during Internet Searching. Am J Geriatr Psychiatry. 2009;17:116-26. 10.1097/JGP.0b013e3181953a02 19155745

[16] Wang R Chen Z Zhou Y Shen L Zhang Z Wu X Melancholy or mahjong? Diversity, frequency, type, and rural-urban divide of social participation and depression in middle- and old-aged Chinese: A fixed-effects analysis. Soc Sci Med. 2019;238:112518. 10.1016/j.socscimed.2019.112518 31473574

[17] Zuelsdorff ML Koscik RL Okonkwo OC Peppard PE Hermann BP Sager MA Social support and verbal interaction are differentially associated with cognitive function in midlife and older age. Neuropsychol Dev Cogn B Aging Neuropsychol Cogn. 2019;26:144-60. 10.1080/13825585.2017.1414769 29241403 PMC6003840

[18] Lee Y Yeung W-JJ Gender matters: Productive social engagement and the subsequent cognitive changes among older adults. Soc Sci Med. 2019;229:87-95. 10.1016/j.socscimed.2018.08.024 30177360

[19] Tomioka K Kurumatani N Hosoi H Social Participation and Cognitive Decline Among Community-dwelling Older Adults: A Community-based Longitudinal Study. J Gerontol B Psychol Sci Soc Sci. 2018;73:799-806. 27194753 10.1093/geronb/gbw059

[20] Croezen S Avendano M Burdorf A van Lenthe FJ Social Participation and Depression in Old Age: A Fixed-Effects Analysis in 10 European Countries. Am J Epidemiol. 2015;182:168-76. 10.1093/aje/kwv015 26025236 PMC4493978

[21] Luo Y Pan X Zhang Z Productive activities and cognitive decline among older adults in China: Evidence from the China Health and Retirement Longitudinal Study. Soc Sci Med. 2019;229:96-105. 10.1016/j.socscimed.2018.09.052 30274688

[22] Lei X Smith JP Sun X Zhao Y Gender differences in cognition in China and reasons for change over time: Evidence from CHARLS. J Econ Ageing. 2014;4:46-55. 10.1016/j.jeoa.2013.11.001 25530942 PMC4269268

[23] China Center for Economic Research Peking University. China Health and Retirement Longitudinal Study followup questionnaire 2015. 2015. Available: http://charls.pku.edu.cn/pages/data/111/zh-cn.html. Accessed.

[24] Lin W A study on the factors influencing the community participation of older adults in China: based on the CHARLS2011 data set. Health Soc Care Community. 2017;25:1160-8. 10.1111/hsc.12415 28178751

[25] Zeng Y Feng QS Hesketh T Christensen K Vaupel JW Survival, disabilities in activities of daily living, and physical and cognitive functioning among the oldest-old in China: a cohort study. Lancet. 2017;389:1619-29. 10.1016/S0140-6736(17)30548-2 28285816 PMC5406246

[26] Lv X Li W Ma Y Chen H Zeng Y Yu X Cognitive decline and mortality among community-dwelling Chinese older people. BMC Med. 2019;17:63. 10.1186/s12916-019-1295-8 30871536 PMC6419492

[27] Livingston G Sommerlad A Orgeta V Costafreda SG Huntley J Ames D Dementia prevention, intervention, and care. Lancet. 2017;390:2673-734. 10.1016/S0140-6736(17)31363-6 28735855

[28] Tomioka K Kurumatani N Hosoi H Positive and negative influences of social participation on physical and mental health among community-dwelling elderly aged 65-70 years: a cross-sectional study in Japan. BMC Geriatr. 2017;17:111. 10.1186/s12877-017-0502-8 28525988 PMC5437627

[29] Thomas PA Trajectories of Social Engagement and Limitations in Late Life. J Health Soc Behav. 2011;52:430-43. 10.1177/0022146511411922 22144732

[30] Sakamoto A Ukawa S Okada E Sasaki S Zhao W Kishi T The association between social participation and cognitive function in community-dwelling older populations: Japan Gerontological Evaluation Study at Taisetsu community Hokkaido. Int J Geriatr Psychiatry. 2017;32:1131-40. 10.1002/gps.4576 27610611

[31] Proulx CM Curl AL Ermer AE Longitudinal Associations Between Formal Volunteering and Cognitive Functioning. J Gerontol B Psychol Sci Soc Sci. 2018;73:522-31. 10.1093/geronb/gbx110 28958028 PMC5927087

[32] Chu-Man L Chang M-Y Chu M-C Effects of mahjong on the cognitive function of middle-aged and older people. Int J Geriatr Psychiatry. 2015;30:995-7. 10.1002/gps.4307 26220879

[33] Tsang WWN Wong GCK Gao KL Mahjong playing and eye-hand coordination in older adults-a cross-sectional study. J Phys Ther Sci. 2016;28:2955-60. 10.1589/jpts.28.2955 27821969 PMC5088160

[34] Cheng ST Chan ACM Yu ECS An exploratory study of the effect of mahjong on the cognitive functioning of persons with dementia. Int J Geriatr Psychiatry. 2006;21:611-7. 10.1002/gps.1531 16779765

[35] Wang CH Tsai CL Tseng P Yang AC Lo MT Peng CK The association of physical activity to neural adaptability during visuo-spatial processing in healthy elderly adults: A multiscale entropy analysis. Brain Cogn. 2014;92C:73-83. 10.1016/j.bandc.2014.10.006 25463141

[36] Domazet SL Tarp J Huang T Gejl AK Andersen LB Froberg K Associations of Physical Activity, Sports Participation and Active Commuting on Mathematic Performance and Inhibitory Control in Adolescents. PLoS One. 2016;11:e0146319. 10.1371/journal.pone.0146319 26727211 PMC4699746

[37] He Q Cui Y Liang L Zhong Q Li J Li Y Social participation, willingness and quality of life: A population-based study among older adults in rural areas of China. Geriatr Gerontol Int. 2017;17:1593-602. 27869351 10.1111/ggi.12939

[38] Ertel KA Glymour MM Berkman LF Effects of social integration on preserving memory function in a nationally representative US elderly population. Am J Public Health. 2008;98:1215-20. 10.2105/AJPH.2007.113654 18511736 PMC2424091

[39] Lu J Fu W Liu Y Physical activity and cognitive function among older adults in China: A systematic review. J Sport Health Sci. 2016;5:287-96. 10.1016/j.jshs.2016.07.003 30356530 PMC6188717

[40] Wang R Bishwajit G Zhou YJ Wu X Feng D Tang SF Intensity, frequency, duration, and volume of physical activity and its association with risk of depression in middle- and older-aged Chinese: Evidence from the China Health and Retirement Longitudinal Study, 2015. PLoS One. 2019;14:e0221430. 10.1371/journal.pone.0221430 31425559 PMC6699736

[41] Lund R Nilsson CJ Avlund K Can the higher risk of disability onset among older people who live alone be alleviated by strong social relations? A longitudinal study of non-disabled men and women. Age Ageing. 2010;39:319-26. 10.1093/ageing/afq020 20208073

[42] Wang H-X Jin Y Hendrie HC Liang C Yang L Cheng Y Late Life Leisure Activities and Risk of Cognitive Decline. J Gerontol A Biol Sci Med Sci. 2013;68:205-13 10.1093/gerona/gls153 22879456 PMC3598354

[43] Guiney H Machado L Volunteering in the Community: Potential Benefits for Cognitive Aging. J Gerontol B Psychol Sci Soc Sci. 2018;73:399-408. 10.1093/geronb/gbx134 29161431

