# Supplementary material for: Association between social participation and cognitive function among middle- and old-aged Chinese: A fixed-effects analysis
Source: J Glob Health. 2020 Sep 15;10(2):020801. doi: 10.7189/jogh.10.020801 (PMC7568928; doi:10.7189/jogh.10.020801)
Supplement: Online Supplementary Document [file jogh-10-020801-s001.pdf]

Online Supplementary Documents

Table S1 Association between social participation and cognitive function using dataset with multiple imputation techniques (n=13,091)

	Total Score			Memory			Mental Status		
	All	Men	Women	All	Men	Women	All	Men	Women
	β (95% CI)	β (95% CI)	β (95% CI)	β (95% CI)	β (95% CI)	β (95% CI)	β (95% CI)	β (95% CI)	β (95% CI)
**Variety (Ref: None)**									
1 type	0.382***<br>(0.272, 0.491)	0.407***<br>(0.247, 0.567)	0.358***<br>(0.208, 0.509)	0.254***<br>(0.167, 0.342)	0.266***<br>(0.139, 0.392)	0.244***<br>(0.123, 0.365)	0.127***<br>(0.067, 0.187)	0.141**<br>(0.052, 0.231)	0.114**<br>(0.034, 0.194)
≥ 2 types	0.673***<br>(0.526, 0.821)	0.600***<br>(0.394, 0.806)	0.753***<br>(0.542, 0.963)	0.453***<br>(0.335, 0.570)	0.406***<br>(0.244, 0.568)	0.501***<br>(0.331, 0.671)	0.221***<br>(0.141, 0.301)	0.194**<br>(0.079, 0.309)	0.251***<br>(0.139, 0.364)
**Frequency (Ref: None)**									
Not regularly	0.324***<br>(0.172, 0.448)	0.341***<br>(0.149, 0.534)	0.307**<br>(0.110, 0.504)	0.208***<br>(0.099, 0.318)	0.206**<br>(0.054, 0.357)	0.212*<br>(0.054, 0.371)	0.115**<br>(0.040, 0.190)	0.136*<br>(0.028, 0.243)	0.095+<br>(-0.010, 0.200)
≥ 1/week	0.531***<br>(0.428, 0.661)	0.540***<br>(0.369, 0.711)	0.524***<br>(0.365, 0.683)	0.360***<br>(0.267, 0.453)	0.370***<br>(0.235, 0.505)	0.351***<br>(0.223, 0.492)	0.171***<br>(0.108, 0.235)	0.170***<br>(0.074, 0.265)	0.193***<br>(0.088, 0.258)
**Interacting with friends (Ref: None)**									
Not regularly	0.334***<br>(0.187, 0.481)	0.326**<br>(0.118, 0.535)	0.341**<br>(0.135, 0.548)	0.208***<br>(0.090, 0.325)	0.221**<br>(0.057, 0.386)	0.196*<br>(0.030, 0.363)	0.126**<br>(0.046, 0.206)	0.105+<br>(-0.012, 0.221)	0.145**<br>(0.035, 0.255)
≥ 1/week	0.441***<br>(0.322, 0.559)	0.475***<br>(0.300, 0.650)	0.419***<br>(0.258, 0.579)	0.294***<br>(0.200, 0.388)	0.339***<br>(0.201, 0.476)	0.265***<br>(0.136, 0.394)	0.147***<br>(0.082, 0.211)	0.136**<br>(0.039, 0.234)	0.154***<br>(0.068, 0.239)
**Mah-jong, cards, chess or other clubs (Ref: None)**									
Not regularly	0.222*<br>(0.024, 0.420)	0.217+<br>(-0.035, 0.469)	0.220<br>(-0.097, 0.537)	0.183*<br>(0.025, 0.341)	0.156<br>(-0.042, 0.355)	0.205<br>(-0.050, 0.460)	0.039<br>(-0.069, 0.147)	0.060<br>(-0.080, 0.201)	0.015<br>(-0.154, 0.184)
≥ 1/week	0.448***<br>(0.251, 0.646)	0.316*<br>(0.060, 0.572)	0.606***<br>(0.300, 0.912)	0.359***<br>(0.202, 0.517)	0.218*<br>(0.016, 0.419)	0.524***<br>(0.278, 0.770)	0.089<br>(-0.018, 0.196)	0.099<br>(-0.044, 0.241)	0.082<br>(-0.081, 0.245)

**Voluntary activities (Ref: None)**									
Not	0.275***	0.210+	0.350**	0.209**	0.172+	0.252*	0.065	0.038	0.097
regularly	(0.112, 0.438)	(-0.012, 0.432)	(0.111, 0.589)	(0.079, 0.339)	(-0.003, 0.347)	(0.059, 0.444)	(-0.023, 0.154)	(-0.086, 0.162)	(-0.029, 0.225)
≥	0.549***	0.775***	0.348+	0.350***	0.431**	0.286+	0.199**	0.343***	0.062
1/week	(0.289, 0.808)	(0.408, 1.142)	(-0.019, 0.715)	(0.142, 0.557)	(0.142, 0.720)	(-0.010, 0.581)	(0.058, 0.340)	(0.139, 0.548)	(-0.133, 0.258)
**Sports or social clubs (Ref: No)**									
Yes	0.281**	0.013	0.479***	0.157+	0.007	0.265*	0.124*	0.005	0.214**
	(0.071, 0.491)	(-0.306, 0.331)	(0.199, 0.759)	(-0.010, 0.324)	(-0.243, 0.258)	(0.040, 0.490)	(0.009, 0.228)	(-0.173, 0.182)	(0.065, 0.363)
**Internet (Ref: No)**									
Yes	0.780***	0.793***	0.769*	0.584***	0.545**	0.656*	0.196+	0.248+	0.114
	(0.409, 1.150)	(0.321, 1.264)	(0.180, 1.359)	(0.288, 0.879)	(0.173, 0.916)	(0.181, 1.130)	(-0.005, 0.397)	(-0.015, 0.511)	(-0.200, 0.428)
**Community organisations (Ref: No)**									
Yes	0.387*	0.107	0.719**	0.146	0.068	0.238	0.241*	0.039	0.482***
	(0.031, 0.743)	(-0.364, 0.579)	(0.182, 1.257)	(-0.138, 0.430)	(-0.303, 0.439)	(-0.195, 0.670)	(0.048, 0.439)	(-0.224, 0.302)	(0.196, 0.768)

N.B. All models controlled time-varying variables, including year, education, marital status, living near children, retirement status, percapita household income, alcohol consumption, smoking, # of types of NCDs, and # of types of lower body constraints; + $P < 0.1$, * $P < 0.05$, ** $P < 0.01$, *** $P < 0.001$.

Table S2 correlation and cross-sectional regression analysis of the relationship between SP and cognitive function

	Correlation			Cross-sectional regression[‡]		
	Total Score	Memory	Mental Status	Total Score	Memory	Mental Status
	rho (z)	rho (z)	rho (z)	β (95% CI)	β (95% CI)	β (95% CI)
**Variety**						
None				1	1	1
1 type	0.195*** (>8.210)	0.172*** (>8.210)	0.150*** (>8.210)	0.641*** (0.482, 0.799)	0.395*** (0.282, 0.508)	0.246*** (0.158, 0.333)
≥ 2 types				1.425*** (1.246, 1.605)	0.873*** (0.744, 1.002)	0.552*** (0.453, 0.652)
**Frequency**						
None				1	1	1
Not regularly	0.164*** (>8.210)	0.146*** (>8.210)	0.125*** (>8.210)	0.679*** (0.478, 0.880)	0.409*** (0.265, 0.553)	0.270*** (0.159, 0.381)
≥ 1/week				1.066*** (0.914, 1.218)	0.658*** (0.549, 0.767)	0.408*** (0.324, 0.492)
**Interacting with friends**						
None				1	1	1
Not regularly	0.088*** (>8.210)	0.089*** (>8.210)	0.052*** (>8.210)	0.539*** (0.325, 0.753)	0.371*** (0.218, 0.524)	0.168*** (0.050, 0.286)
≥ 1/week				0.713*** (0.551, 0.874)	0.473*** (0.358, 0.589)	0.239*** (0.150, 0.329)
**Mah-jong, cards, chess or other clubs**						
None				1	1	1
Not regularly	0.141*** (>8.210)	0.105*** (>8.210)	0.131*** (>8.210)	0.911*** (0.666, 1.157)	0.469*** (0.293, 0.644)	0.443*** (0.307, 0.578)
≥ 1/week				1.011*** (0.805, 1.218)	0.580*** (0.432, 0.728)	0.431*** (0.317, 0.545)
**Voluntary activities**						
None				1	1	1
Not regularly	0.090*** (>8.210)	0.091*** (>8.210)	0.055*** (>8.210)	0.770*** (0.532, 1.008)	0.468*** (0.298, 0.639)	0.302*** (0.170, 0.433)
≥ 1/week				0.690*** (0.317, 1.063)	0.599*** (0.333, 0.866)	0.091 (-0.115, 0.296)
**Sports or social clubs**[†]						
No				1	1	1
Yes	-2.488*** (-22.002)	-1.481*** (-18.625)	-1.007*** (-17.309)	1.032*** (0.772, 1.292)	0.678*** (0.492, 0.864)	0.354*** (0.211, 0.497)
**Internet**[†]						
No				1	1	1

Yes	-5.201*** (-38.456)	-3.107*** (-28.038)	-2.094*** (-33.305)	1.721*** (1.283, 2.158)	1.235*** (0.923, 1.548)	0.485*** (0.244, 0.726)
**Community organisations**†						
No				1	1	1
Yes	-2.927*** (-14.750)	-1.653*** (-11.709)	-1.274*** (-13.036)	1.303*** (0.838, 1.768)	0.857*** (0.524, 1.189)	0.447*** (0.191, 0.703)

N.B. †: results from t test in a form of difference in mean value (t); ‡: the cross-sectional regression analysis was based on pooled data from 2011, 2013 and 2015. All regression models controlled time-varying and time-invariant variables, including gender, residency, year, age, education, marital status, living near children, retirement status, percapita household income, alcohol consumption, smoking, # of types of NCDs, and # of types of lower body constraints; + $P < 0.1$, * $P < 0.05$, ** $P < 0.01$, *** $P < 0.001$.
